# Breast cancer: miRNAs monitoring chemoresistance and systemic therapy

**DOI:** 10.3389/fonc.2023.1155254

**Published:** 2023-06-16

**Authors:** Shivam Singh, Heena Saini, Ashok Sharma, Subhash Gupta, V. G. Huddar, Richa Tripathi

**Affiliations:** ^1^ Department of Radiation Oncology, Dr. B. R. Ambedkar Institute Rotary Cancer Hospital, All India Institute of Medical Sciences, New Delhi, India; ^2^ Integrated translational Molecular Biology laboratory, Department of Rog Nidan and Vikriti vigyan (Pathology), All India Institute of Ayurveda (AIIA), New Delhi, India; ^3^ Department of Biochemistry, All India Institute of Medical Sciences, New Delhi, India; ^4^ Department of Kaya Chikitsa (Internal Medicine), All India Institute of Ayurveda (AIIA), New Delhi, India

**Keywords:** micro RNA, breast cancer, chemoresistance, neoadjuvant (chemo)radiotherapy, systemic therapies

## Abstract

With a high mortality rate that accounts for millions of cancer-related deaths each year, breast cancer is the second most common malignancy in women. Chemotherapy has significant potential in the prevention and spreading of breast cancer; however, drug resistance often hinders therapy in breast cancer patients. The identification and the use of novel molecular biomarkers, which can predict response to chemotherapy, might lead to tailoring breast cancer treatment. In this context, accumulating research has reported microRNAs (miRNAs) as potential biomarkers for early cancer detection, and are conducive to designing a more specific treatment plan by helping analyze drug resistance and sensitivity in breast cancer treatment. In this review, miRNAs are discussed in two alternative ways-as tumor suppressors to be used in miRNA replacement therapy to reduce oncogenesis and as oncomirs to lessen the translation of the target miRNA. Different miRNAs like miR-638, miR-17, miR-20b, miR-342, miR-484, miR-21, miR-24, miR-27, miR-23 and miR-200 are involved in the regulation of chemoresistance through diverse genetic targets. For instance, tumor-suppressing miRNAs like miR-342, miR-16, miR-214, and miR-128 and tumor-promoting miRNAs like miR101 and miR-106-25 cluster regulate the cell cycle, apoptosis, epithelial to mesenchymal transition and other pathways to impart breast cancer drug resistance. Hence, in this review, we have discussed the significance of miRNA biomarkers that could assist in providing novel therapeutic targets to overcome potential chemotherapy resistance to systemic therapy and further facilitate the design of tailored therapy for enhanced efficacy against breast cancer.

## Highlights

1. Drug resistance is the major obstacle in breast cancer chemotherapy
2. miRNA biomarkers could provide novel therapeutic targets to overcome potential chemotherapy resistance to systemic therapy in breast cancer.
3. Targeting miRNAs—either reducing or raising their expression—seems to be an attractive approach for designing novel, more effective, and personalized treatments for breast cancer.
4. A novel approach to treating breast cancer that combines miRNA therapies with conventional chemotherapeutic techniques and drug targets is possible.

## 1 Introduction

The most common malignancy worldwide is breast cancer (BC). According to the status update on the GLOBOCAN 2020 projections of cancer incidence and mortality, BC is the primary cause of cancer death in women and accounts for 1 in 4 cancer diagnoses among females (1). An estimated 684,996 people died from breast cancer in 2020, with low-resource areas accounting for a disproportionate share of these deaths. According to statistics, the prevalence of BC ranges from 2-6% in Western nations to 10-20% in Asian nations (2), indicating that BC is becoming a global health concern, even in nations with sizable young populations like India. Although breast cancer diagnoses have increased recently (3), the prognosis for the disease has significantly improved, with expected 5-year survival rates rising from 40% to approximately 90% over the last 50 years. With few exceptions, en-bloc radical resections in the form of Halstead mastectomy and axillary clearing were formerly thought to be essential for managing BC (4). Recent advancements in clinical trials have been brought about by a greater understanding of the molecular processes associated with the heterogeneity of breast tumors. This understanding has allowed for more conservative surgical procedures and the personalization of treatment plans to maximize sensitization to the tumor while minimizing unneeded morbidity to the patient. This includes the era of cancer diagnostics, which has recognized BC as a diverse disease and routinely subcategorized these cancers into four genetically distinct, integral subgroups - luminal A breast cancer (LABC), luminal B breast cancer (LBBC), human epidermal growth factor receptor-2 enriched breast cancer (HER2+) and triple-negative breast cancer (TNBC). These subgroups have different clinical behavior, prognosis, treatment approaches, and clinical outcomes in the known treatments (5).

For BC patients, chemotherapy is regarded as the most successful and crucial therapeutic approach. Anthracyclins, Tamoxifen, Taxane, 5-FU and trastuzumab are the major chemotherapeutic drugs which are administered to BC patients (6–12). Doxorubicin, daunorubicin, and epirubicin are some of the anthracycline antibiotics that are frequently used. Anthracyclines can be administered at all BC stages and have demonstrated a crucial function in treating BC (8). Tamoxifen is specifically used for the oestrogen receptor (ER) positive subtype of BC (9). Anthracyclines and taxanes are used as the predominant treatment for TNBC (10). The two most widely used taxanes are- paclitaxel and docetaxel, causing acute hypersensitivity responses (HSRs) in 5% to 10% of patients (11). The human epidermal growth factor receptor 2 (HER-2) is frequently used to categorize BC patients based on its overexpression (also known as HER-2 positive) or lack of expression (also known as HER-2 negative) (13). The likelihood of BC metastases and poor prognosis are strongly correlated with HER-2 overexpression (13). A targeted therapy for HER-2 is trastuzumab (TRS), a humanized monoclonal antibody (12). Despite our efforts to categorize tumors into prognostic categories, tumor behavior and prognosis remains unpredictable, which makes it challenging to develop strategies that would improve disease control while minimizing toxicities to patients. Although, a better understanding of the disease has led to advancements in treatment over the past few decades, but drug resistance remains a challenge and the underlying molecular causes are still largely undefined (14). Drug-resistant cancer cells multiply rapidly and grow more hostile, increasing the likelihood that the tumor may aggressively spread to other organs. Drug resistance can be categorized in two different ways. One is internal resistance or inherited resistance, which occurs when tumors are resistant to treatment even before receiving it, meaning that even early detection and treatment are ineffective. Another form of resistance is received resistance or acquired resistance which occurs following an initial positive response to the therapy (15). Here, the targets and processes associated are a focus of significant research, and the mechanisms of such drug resistance are largely still under investigation (16). For instance, Martz et al. (2014) demonstrated that stimulation of the Notch-1, mitogen-activated protein kinase (RAS-MAPK), phosphoinositide 3-kinase (PI3K) and mammalian target of rapamycin (mTOR), PI3K/AKT and estrogen receptor (ER) signaling pathways resulted in resistance to a variety of drugs (17). It was observed that when Notch-1 is activated, BRAF (V600E) melanoma cells develop acquired resistance to MAPK inhibitors and breast cancer cells also exhibit resistance to tamoxifen (17). Hence, the research group used a Notch-1 inhibitor to restore sensitivity, indicating that Notch-1 knockdown could be a therapeutic strategy in melanomas and drug-resistant breast malignancies (17). Likewise, it seems, resistance to chemotherapy is also related to the epidermal growth factor receptor (EGFR) pathway. Genetically modified murine model (GEMM), human cell lines, and a clinically applicable model of KRAS-mutant colorectal cancer (CRC) have all been used to study EGFR and PI3K/mTOR (18). According to the evidence, PI3K/mTOR and EGFR inhibition boost drug sensitivity and are increasingly used in cancer therapy to combat drug resistance. Additionally, the use of systemic drugs as neoadjuvant enables the production of *in-vivo* data on tumor sensitivity, which has been shown to have predictive importance for disease survival and recurrence. These contemporary aspects of traditional breast cancer management shed light on the potential value of emerging biomarkers in advancing the current treatment model. There are currently few biomarkers that may reliably predict response and resistance to systemic and targeted therapy and attempts to use non-invasive approaches to collect such biomarkers have largely been ineffective (19). This highlights how important it is for researchers to find new biomarkers that can assess patient response to therapy, predict the prognosis of breast cancer patients, and offer clinicians cutting-edge oncogenesis-targeting therapeutic approaches. In the context of BC chemoresistance monitoring and systemic therapy, this study focuses on the function of microRNA (miRNA) as new clinical biomarkers.

miRNAs are small non-coding RNAs ranging from 19 to 25 nucleotides in size and are involved in a variety of biological activities, including cell cycle, apoptosis, survival, and gene control (20). miRNAs primarily bind to the 3′ or 5′ untranslated region (UTR) of their target mRNAs and, depending on the degree of binding, participate in controlling the translation of proteins or destruction of the mRNA itself (21). A single miRNA may target several mRNAs, while many miRNAs may target single mRNA with varying degrees of efficiency (22). Therefore, changes in miRNA expression levels and gene expression silencing by miRNAs have a significant impact on human health and the emergence of diseases such as cancer, diabetes, neurological disease, and cardiovascular disorders (23–25). In the context of cancer, miRNAs can function as both tumor suppressors and oncogenes/oncomirs (26). In contrast to their counterparts in normal tissue, many miRNAs are reported to be up-or down-regulated in cancer tissues. For instance, practically all cancer types have increased miR-21 expression (27). Numerous B-cell malignancies have been shown to express miR-155 at high levels (28). One of the first miRNAs to be found was let-7 which is essentially missing throughout embryonic stages or tissues, although it is highly expressed in the majority of differentiated tissues (29). Similar to the fall in let-7 expression during development, the decline in let-7 expression in malignancies is more pronounced in cancer cells that are more advanced, less differentiated and have mesenchymal features (29). The generation, biology and function of miRNAs in cancer have been discussed in detail in further sections.

This review focuses primarily on the latest findings about the involvement of miRNAs in breast tumor resistance to chemotherapeutic agents and in the development of systemic therapy. Targeting miRNAs—either reducing or raising their expression—seems to be an attractive approach for designing novel, more effective, and personalized treatments for BC. Boosting drug efficacy by examining the downstream targets/pathways influenced by miRNA targeting and predicting patient response to various therapies can lead to better treatment outcomes for BC patients.

## 2 Breast cancer chemotherapy

Breast cancer bears 7% of the total number of cancers related deaths in 2020. To date, many strategies have been adapted to combat this disease. Complete surgical removal has usually enabled efficient breast cancer disease management (30). Regardless of the severity of the disease, William Halstead’s radical mastectomy (which required significant removal of all the breast parenchyma, local lymph nodes, and pectoralis major muscle) used to be the cornerstone of breast cancer treatment (4). Cyclophosphamide, methotrexate, and 5-fluorouracil (CMF), the first chemotherapeutic treatment prescribed by Bonadanno et al. in 1976 with the intention of curing breast cancer, significantly decreased breast cancer relapse (94.7% of 207 patients administered chemotherapy *vs* 76.0% of 179 patients constrained chemotherapy) (31). However, since 1950s, Bernard Fisher and the National Surgical Adjuvant Breast and Bowel Project (NSABP) have hypothesized that aggressive surgery for breast cancer has only limited scientific and biomolecular justification because it is frequently insufficient to achieve complete disease control (32). Fisher’s theory that all breast cancer patients needed systemic therapy (especially with chemotherapy) has, however, been thoroughly refuted.

However, the inherent advantage of treating cancer patients with chemotherapy in the neoadjuvant setting has now been recognized as an oncological practice. Neoadjuvant chemotherapy (NAC) benefits comprised tumor downstaging, expanding patient suitability for breast conservation surgery (BCS), and producing *in vivo* data related to tumor resistance, which has been shown to hold predictive value for cancer recurrence and overall survival (OS) (33, 34). The Early Breast Cancer Triallist’s Collaborative Group (EBCTCG) recently published data from a meta-analysis of randomized clinical trials showing that locoregional recurrence (LRR) rates are higher following neoadjuvant therapy (21.4% *vs*. 15.9%), despite the fact that disease-free survival (DFS) and overall survival (OS) results are parallel with those treated in the adjuvant setting (35). Additionally, there is growing proof that people who have a pathological complete response (pCR) with NAC have a higher chance of living longer than those who have a latent disease (34, 36). Nevertheless, the clinical usefulness of NAC has been integrated into best-practice guidelines for HER2+ and Triple-negative breast cancer (TNBC). HER2+ malignancies should be treated with NAC and trastuzumab, with the exception of T1a-T1b N0 disease (37). High-risk LN (lymph node)- patients and those with LN positivity should receive anthracycline- and taxane-based chemotherapy along with trastuzumab (37). Further, until TNBC is identified with cancer stages T1a-T1b N0, patients with TNBC should always be provided with an anthracycline and taxane-based treatment (37). American Society of Clinical Oncology (ASCO) also supports the inclusion of platinum-based chemotherapy in TNBC based on the results of a recent meta-analysis because of a higher likelihood to obtain pCR (52.1% versus 37.0%) (38). Pembrolizumab and NAC significantly increased the pCR rates in the KEYNOTE522 trial’s preliminary findings (pembrolizumab and NAC: 64.8% versus placebo and NAC: 51.2%) (39).

## 3 Need for miRNA-based therapy

The idea of improving pCR rates, simplifying the de-escalation of adjuvant therapy post pCR, and minimising treatment-related toxicities for patients receiving these neoadjuvant medicines are the main directions for translational research efforts in the future (40). Therefore, numerous clinical trials have focused on practices that improve pCR rates (40). To further improve the pCR, the idea of manipulating treatment with miRNA-based therapies may be helpful in boosting pCR rates to NAC in breast cancer is now popular, and the same has been discussed in depth in this review.

## 4 Chemoresistance in breast cancer

**Figure 1:**
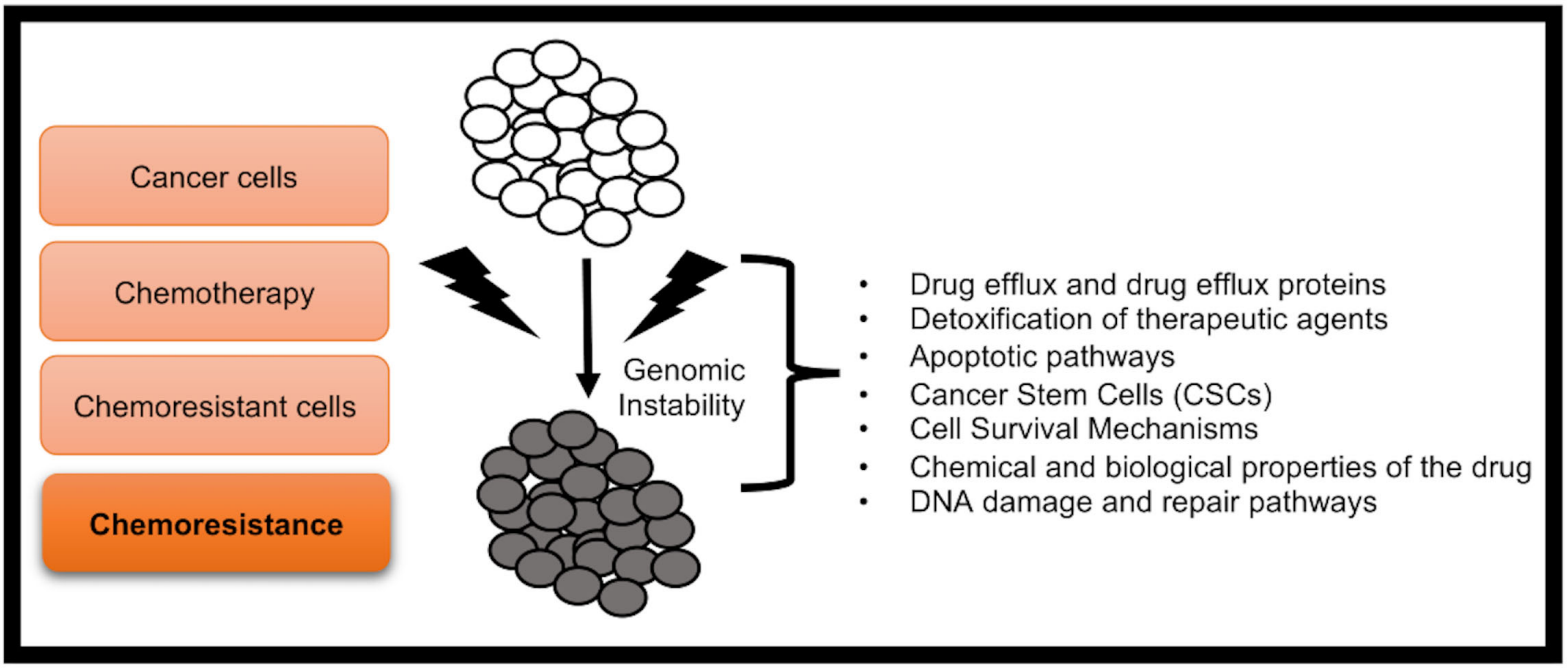
The major chemoresistance mechanisms of cancer cells.

Various molecular aspects are known to be involved in inducing chemoresistance in cancer cells (**Figure 1**). Some of them have also been summarized below:

♦ Resistant genes:

### 4.1 Twist

Twist is a key player in the invasion and metastasis of tumors because it regulates the epithelial-mesenchymal transition (EMT) (41). It has been reported that NF-κB up-regulation of twist-1 is a factor in the chemoresistance (42). Through the downregulation of estrogen receptor alpha (ERα) activity, twist overexpression can also contribute to hormone resistance in breast tumors (43).

### 4.2 Multidrug resistance gene

MDR1 has a significant impact on breast cancer’s chemoresistance. P-glyocoprotein (P-gp), glutathione S-transferase (GST-π), and P53 are only a few examples of MDR1-encoded proteins that are involved in the chemoresistance cascade (44). In fact, Chen et al., 2022 suggested the synergistic effect of 7-*O*-geranylquercetin and miR-451 on undoing MDR-1 and P-gp-mediated chemoresistance in breast cancer MCF-7/ADR (Adriamycin) cells (45).

♦ Efflux proteins: Another mechanism of resistance to chemotherapy is mediated by ATP-dependent efflux pumps, which decrease the intracellular concentration of drugs. By using the energy from ATP hydrolysis, or the MDR phenomenon, the ATP-dependent efflux transporters in cancer cells can actively transport a range of substrates outside the cell membrane (46). MDR-associated proteins, such as P-gp, multidrug resistance-associated protein (MRP), ABCC subfamily, and breast cancer resistance protein (BCRP) are examples of ATP-binding cassette (ABC) transporters (47). Downregulation of ABCC4 with miR-124-3p overexpression significantly enhanced the ADR sensitivity in MCF7/ADR cells (48), highlighting the importance of miRNA-based targeting of resistant proteins for improved treatment of breast tumors that are resistant to certain drugs.
♦ *Signaling pathways:* Resistance to endocrine therapy for breast cancers can be brought on by cell surface receptors like epidermal growth factor receptor (EGFR), human epidermal growth factor receptor-2 (HER-2), and insulin-like growth factor 1 receptor (IGF-1R), and their downstream signaling pathways like PI3K/AKT/mTOR, RAS/MAPK/ERK, and upstream signaling pathways (49). Also, tamoxifen resistance has been linked to EGFR, HER-2/nue, PI3K, and other growth factor signaling pathways (50). Additionally, one of the gefitinib resistance mechanisms in breast cancer may involve EGFR nuclear translocation (51).
♦ *Response to DNA damage:* Many chemotherapeutic medications work to treat cancer by causing DNA damage (52). Single and double-strand breaks, intra- and inter-strand DNA crosslinks, methylated and oxidized bases, mismatched and protein-DNA adducts are several types of DNA damage. Cancer stem cells (CSCs), in particular, stimulate DNA damage repair (DDR) pathways to counteract DNA damage, explaining why chemotherapy that damages DNA might cause drug resistance (53). The following DDR pathways are found in breast cancer cells: homologous recombination (HR) (54) and non-homologous end joining (NHEJ) (55), involved in the elimination of double-strand breaks; mismatch repair (MMR), in charge of removing incorrect bases (56); nucleotide excision repair (NER) (57) and base excision repair (BER) (58), involved in the repair of single-strand breaks. In addition, these pathways contain a variety of components that are involved in the repair procedure, including the DNA repair genes - Excision Repair Cross-Complementation group (ERCC1/2/5) (59, 60), the BRCA1/2 (61), the Meiotic Recombination 11 (MRE11) and RAD50/51 (60, 62), and others. Understanding DNA repair mechanisms can aid in reversing breast cancer resistance to therapy.
♦ *Cancer Stem Cells (CSCs):* Chemoresistance in breast cancer is significantly influenced by cancer stem cells (63). CSCs overexpress several ABC transporters, such as ABCB5, ABCC1, ABCG2, and P-gp. Additionally, CSCs can cause drug resistance through increased anti-apoptotic levels and DNA repair activity (64). Another significant factor contributing to CSCs’ drug resistance is Aldehyde dehydrogenase 1 (ALDH1) upregulation (65). Drug resistance of CSCs may also be influenced by signal transduction pathways such as Notch, Hedgehog, and Wnt/-catenin which are involved in the self-renewal and maintenance of stem cells (66). Expression of Let-7, a tumor suppressor miRNA, was shown to be considerably reduced in breast cancer stem cells compared to non-cancer stem cells. However, the upregulation of let-7 microRNA in breast cancer stem cells can encourage CSCs to enter the differentiation phase from the stationary phase, and hence increases the sensitivity of CSCs to the chemotherapeutics (67).
♦ *Drug detoxification:* Cell detoxification proteins such as ALDH, DNA topoisomerase, protein kinase C, dihydrofolate reductase, glutathione (68) and glutathione S-transferases (GST) are some of the key enzymes that contribute to MDR in cancer cells. These agents have the potential to enhance the transformation and catabolism of anti-neoplastic drugs, shorten the term of effective concentration of chemotherapeutic drugs in tumor cells, decrease drug accumulation in target areas, and ultimately limit drug efficacy (69). For example, GST-π can be employed as a separate index to direct a clinical treatment against BC as its expression in breast cancer patients was associated with the histological grade, the number of lymphatic metastases, and the age of the patients (70).

## 5 MicroRNAs

**Figure 2:**
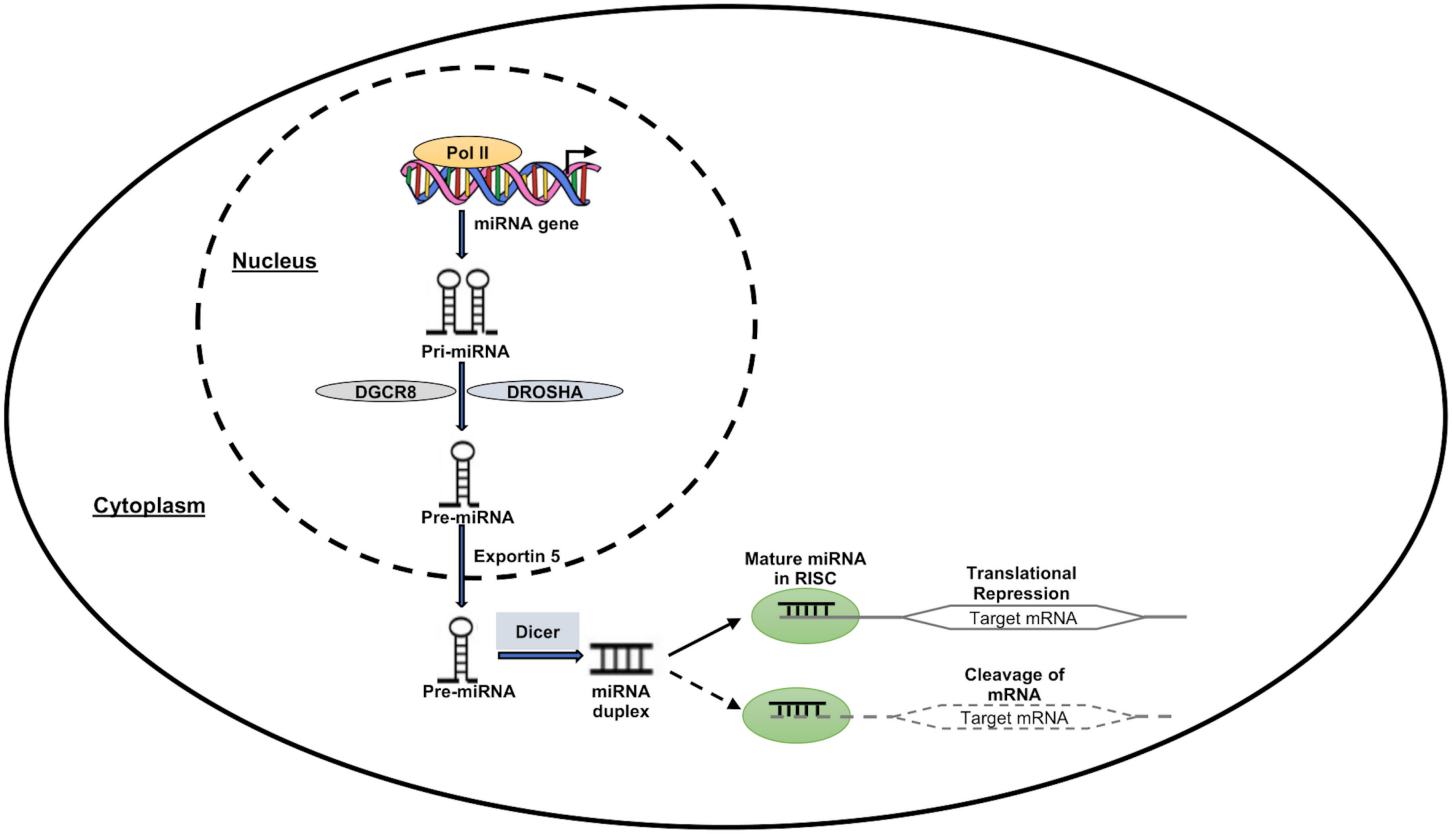
MiRNA expression and function. RNA polymerase II enzyme in the nucleus transcribes the miRNA-encoding genes, forming the “Pri-miRNA” hairpin-shaped molecule. DROSHA and DGCR8 molecules work together to transform the “Pri-miRNA” molecule into the “Pre-miRNA” precursor molecule. Pre-miRNA then travels to the nuclear export receptor “Exportin 5” and reaches the cytoplasm. This precursor is cleaved by Dicer complex in the cytoplasm to create a double-stranded molecule called “miRNA duplex.” One of these two strands is left active after this process, and it has the ability to suppress or even activate the target downstream genes at the transcriptional or translational level.

MicroRNAs are a class of small noncoding RNAs (ncRNAs), which function in post-transcriptional regulation of gene expression and are powerful regulators of various cellular activities and have been linked to many diseases (20). RNA polymerase II (Pol II), which produces the main transcripts, participates in several stages of microRNA synthesis (pri-miRNA). The pri-miRNA are split up into precursor miRNAs (pre-miRNAs) by the RNase III Drosha (71). The pre-miRNAs are subsequently moved from the nucleus into the cytoplasm by Exportin-5 (Exp5), where they are further split by Dicer into a mature single-stranded miRNA. The miRNA is induced to either degrade or suppress the translation of mRNA targets when the mature miRNA is removed from the pre-miRNA hairpin and attached to the RNA-induced silencing complex (RISC) (72). **Figure 2** depicts a pictorial representation of miRNA biogenesis and function. Besides miRNAs, other ncRNAs are long-chain non-coding RNAs (LncRNAs), piRNAs, and circle RNAs (circRNAs), which make up just 1% of the whole genome’s RNA (73).

The molecular revolution enables us to design strategies to maximize patient outcomes, reduce toxicity, and control disease with less strenuous and more focused therapies. The development of chemotherapeutic response biomarkers is necessary in the future to speed up the removal of tumors and reduce the need for extended and excessive treatments. The utility of detecting miRNA expression (both in tumors and in the blood) is now being discussed in the scientific community. Doing so may help doctors prescribe medicines that are suitably targeted, address early relapse, or even enable miRNA-directed therapies. In general, miRNAs can be either tumor suppressors (tumor suppressor miRNA) or oncomirs, and they can affect the development of cancers in either way. Numerous miRNAs, along with their downstream targets, have been shown to be differentially expressed in breast cancer patients when compared to healthy controls (either circulating or in tumors) (**Table 1**).

**Table 1:** miRNA targets and signaling pathways in Breast cancer.

miRNAs	Targets	Functional pathways	References
Tumor suppressor miRNAs			
miR-206	ESR1	ER signaling	(74)
miR-17-5p	AlB1,CCND1,E2F1	Proliferation	(75)
miR-125a,b	HER2,HER3	Anchorage-dependent growth	(76, 77)
miR-200c	BMl1,ZEB1,ZEB2	TGF-beta signaling	(78)
let-7	H-RAS, HMGA2, LIN28, PEBP1	Proliferation, differentiation	(79)
miR-34a	CCND1, CDK6, E2F3, MYC	DNA damage, proliferation	(80)
miR-31	FZD3, ITGA5, M-RIP, MMP16, RDX, RHOA	Metastasis	(81)
miR-335	SOX4, PTPRN2, MERTK, TNC	Metastasis	(82)
miR-27b	CYP1B1	Modulation of the response of tumor to anti-cancer drugs	(83)
Oncogenic miRNAs			
miR-21	BCL2, TPM1, PDCD4, PTEN, MASPIN	Apoptosis	(84)
miR-155	RHOA	TGF-beta signaling	(85)
miR-10b	HOXD10	Metastasis	(86)
miR-373/520c	CD44	Metastasis	(87)
miR-27a	Zinc finger ZBTB10, Myt-1	Cell cycle progression G2-M checkpoint regulation	(88)
miR221/222	p27/Kip1	Tamoxifen resistance	(89)

## 6 miRNAs in BC subtyping

The three main subtypes of breast cancer that exist are (1) Positive ER and PR (2); HER-2 positive; and (3) (ER, PR, and HER-2 negative) Triple negative. However, this subtyping is expanded to a more precise one using the microarray approach for identifying miRNA profiles, including (1):Luminal A (ER-positive with low grade) (2); Luminal B (ER-positive with high grade) (3); HER-2 positive (4); Basal-like (Almost equal to triple-negative condition).

There are several miRNAs that are used for the breast cancer subgrouping as shown in **Table 2** (87). Currently, it is possible to use miRNA profiling for the subgrouping of breast tumors. This capability can therefore aid in the selection of cancer patients who will get adjuvant therapy. In addition, miRNA profiling can be successful in identifying new therapeutic targets by revealing the genetic underpinnings of distinct subgroups of breast cancer.

**Table 2:** MiRNAs used in breast cancer subtyping.

Breast cancer subtypes	miRNA changes
Luminal A	Overexpression of miR-126, miR-136, miR-100, miR-99a, miR-145, miR-10a, miR-199a/b, miR-130a, miR-30a-3p, miR-30a-5p, miR-224, miR-214, let-7a/b/c/f, miR-342
Luminal B	Overexpression of miR-106a/b, miR-93, miR-25, miR-10a, miR-30a-3p, miR-30a-5p, miR-224, let-7b/c/f, miR-342c
HER-2 positive	Overexpression of miR-150, miR-142-3p, miR-142-5p, miR-148a, miR-106b, miR-93, miR-155, miR-25, miR-187
TNBC	Downregulation of miR-139-5p, -10b-5p and -486-5p and up-regulation of miR-455-3p, miR-107, miR-146b-5p, miR-324-5p and miR-20a-5p

## 7 Role of miRNA in BC drug resistance

It is well known that miRNAs can regulate drug resistance to traditional chemotherapeutic medicines, endocrine hormone treatments, and radiotherapies in cancer cells (90–93). It has been shown that miRNA expression can influence a breast cancer patient’s ability to respond to or reject systemic treatment as shown in **Table 3**. The miRNAs along with other ncRNAs significantly reverse the BC cell drug resistance by suppressing signaling pathways such as Wnt/β-catenin, Hippo, AKT, TGF-β, or mTOR signaling pathways. A summary of molecules which can participate in target diversity in miRNA interactions leading to drug resistance using different chemotherapeutic drugs is mentioned in **Table 3**. According to various reports, there are scientific explanations and processes for chemotherapeutic resistance, including altered drug-target interactions, lower active drug doses, and increased tumor tissue survival (143). Numerous miRNA expression profiles have been linked to the prediction of chemoresistance and their regulatory role in influencing chemoresistance to chemotherapeutics. For example, reduced expression of miR-18a, miR-1207-5p, and miR-5195-3p in TNBC has recently been linked to translational research studies that predict resistance to paclitaxel or docetaxel in TNBC (144, 145). Similarly, Wu et al., 2019 discovered that by downregulating the expression of dCMP deaminase (DCTD) in TNBC, upregulation of miR-620 improves tumor resistance to gemcitabine-based chemotherapies (146). Further, finding higher levels of circulating miR-125b in 56 patients with invasive ductal carcinoma receiving curative treatment was associated with chemoresistance (p = 0.008) (147). Hypoxia-inducible factor-1 (HIF-1) pathway-dependent upregulation of cell resilience to hypoxia and inhibition of chemotherapy-induced apoptosis are two mechanisms through which miR-24 has been demonstrated to promote chemoresistance in early breast cancer (148). miR-155 has shown to be linked to drug resistance and cancer development (149) via modulation of FOXO3a signaling, the interruption of TGF-beta, and the induction of drug resistance through RhoA signaling. Likewise, in 25 breast cancer samples, miR-221 has been shown to alter the PTEN/Akt/mTOR signaling pathway, which promotes breast cancer resistance to Adriamycin (150).

**Table 3:** ncRNAs related to drug resistance in Breast cancer.

ncRNA	Drugs	Function	Targets/mechanisms	References
miR-200b/c	Tamoxifen	Sensitivity	Activation of vimentin/ZEB/EMT	(94)
miR-186-3p	Tamoxifen	Resistance	Activation of EREG/EGFR	(95)
miR-221/222	Tamoxifen	Resistance	Inhibition of p27Kip1	(89)
miR-449a	Tamoxifen	Sensitivity	Inhibition of ADAM22	(96)
miR-451a	Tamoxifen	Sensitivity	Inhibition of MIF	(97)
lncRNA-ADAMTS9-AS2	Tamoxifen	Sensitivity	Inhibition of PTEN	(98)
lncRNA-ROR	Tamoxifen	Resistance	Inhibition of EMT	(99)
circRNA-0025202	Tamoxifen	Sensitivity	Inhibition of FOXA3a	(100)
miR-200c	Doxorubicin	Sensitivity	Inhibition P-gp	(101)
miR-34a	Adriamycin	Sensitivity	Inhibition Notch1	(102)
miR-302a/b/c/d	Adriamycin	Sensitivity	Activation P-gp MAPK/ERK	(103)
miR-148/152	Adriamycin	Resistance	Inhibition SPIN1	(103)
miR-124-3p	Adriamycin	Sensitivity	Inhibition ABCC4	(48)
miR-298	Adriamycin	Resistance	Inhibition P-gp	(104)
miR-29a	Adriamycin	Resistance	Inhibition PTEN/AKT/GSK3β	(105)
miR-130b	Adriamycin	Resistance	Inhibition PI3K/AKT	(106)
miR-222	Adriamycin	Resistance	Inhibition PTEN/AKT/p27 KIP1	(107)
miR-145	Doxorubicin	Sensitivity	Inhibition MRP1	(108)
miR-489	Adriamycin	Sensitivity	Inhibition EMT/Smad3	(109)
miR-760	Doxorubicin	Resistance	Inhibition EMT/Nanog	(110)
miR-192-5p	Doxorubicin	Sensitivity	Activation JNK/Bad/Caspase9, inhibition Bcl-2/PPIA	(111)
miR-221	Adriamycin	Sensitivity	Inhibition hormone receptor(HR)	(112)
LncRNA-00518	Adriamycin	Resistance	Inhibition miR-199a/MRP1 axis	(113)
Lin28	Paclitaxel	Resistance	Activation of p21 and Rb; inhibition of Let-7	(114)
Let-7a	Paclitaxel	Resistance	Inhibition of caspase-3	(115)
miR-125b	Paclitaxel	Resistance	Inhibition of BAK1	(76)
miR-520h	Paclitaxel	Resistance	Inhibition of DAPK2	(116)
miR-451	Paclitaxel	Resistance	Inhibition of Bcl-2	(117)
miR-100	Paclitaxel	Sensitivity	Inhibition of the Mtor signaling pathway	(118)
miR-18a	Paclitaxel	Resistance	Inhibition of the mTOR signaling pathway	(119)
miR-101	Paclitaxel	Sensitivity	Inhibition of MCL-1	(120)
LncRNA-CASC2	Paclitaxel	Resistance	Inhibition miR-18a-5p/CDK19	(121)
miR-141	Docetaxel	Sensitivity	Activation of EIF4E/CP110	(122)
miR-129-3p	Docetaxel	Resistance	Inhibition of CP110	(123)
miR-3646	Docetaxel	Resistance	Activation of the GSK-3β/β-catenin signaling pathway	(124)
miR-452	Docetaxel	Resistance	Inhibition of APC4	(125)
miR-663	Docetaxel	Resistance	Inhibition of HSPG2	(126)
miR-139-5p	Docetaxel	Resistance	Inhibition of Notch1	(127)
miR-125a-3p	Docetaxel	Sensitivity	Inhibition of BRCA1	(77)
miR-222/29a	Docetaxel	Resistance	Activation of Akt/mTOR	(128)
LncRNA-EPB41L4A-AS2	Docetaxel	Sensitivity	Activation of ABCB1	(129)
miR-125a	Fluorouracil	Resistance	Inhibition LIF/Hippo signaling pathway	(130)
miR-508-5p	Fluorouracil	Resistance	Inhibition P-gp or ZNRD1	(131)
miR-200/203	Fluorouracil	Sensitivity	Inhibition P53/Bmi1	(132)
miR-448	Fluorouracil	Resistance	Inhibition EMT/NFkB	(133)
LncRNA-NEAT1	Fluorouracil	Resistance	Inhibition miR-211/HMGA2	(134)
Circ-CDR1as	Fluorouracil	Resistance	Inhibition miR-7/CCNE1	(135)
miR-21	Trastuzumab	Resistance	Inhibition of AKT and NF-κB	(84)
miR-221	Trastuzumab	Resistance	Inhibition of PTEN	(136)
miR-200c	Trastuzumab	Resistance	Inhibition of ZNF217/ZEB1/TGF-β signaling pathway	(137)
miR-375	Trastuzumab	Sensitivity	Inhibition of IGF1R	(138)
miR-542-3p	Trastuzumab	Sensitivity	Activation of PI3K/AKT	(139)
miR-630	Trastuzumab	Sensitivity	Inhibition of IGF1R	(140)
miR-16	Trastuzumab	Sensitivity	Inhibition of CCNJ and FUBP1	(141)
miR-7	Trastuzumab	Resistance	Inhibition of EGFR	(142)

**Figure 3:**
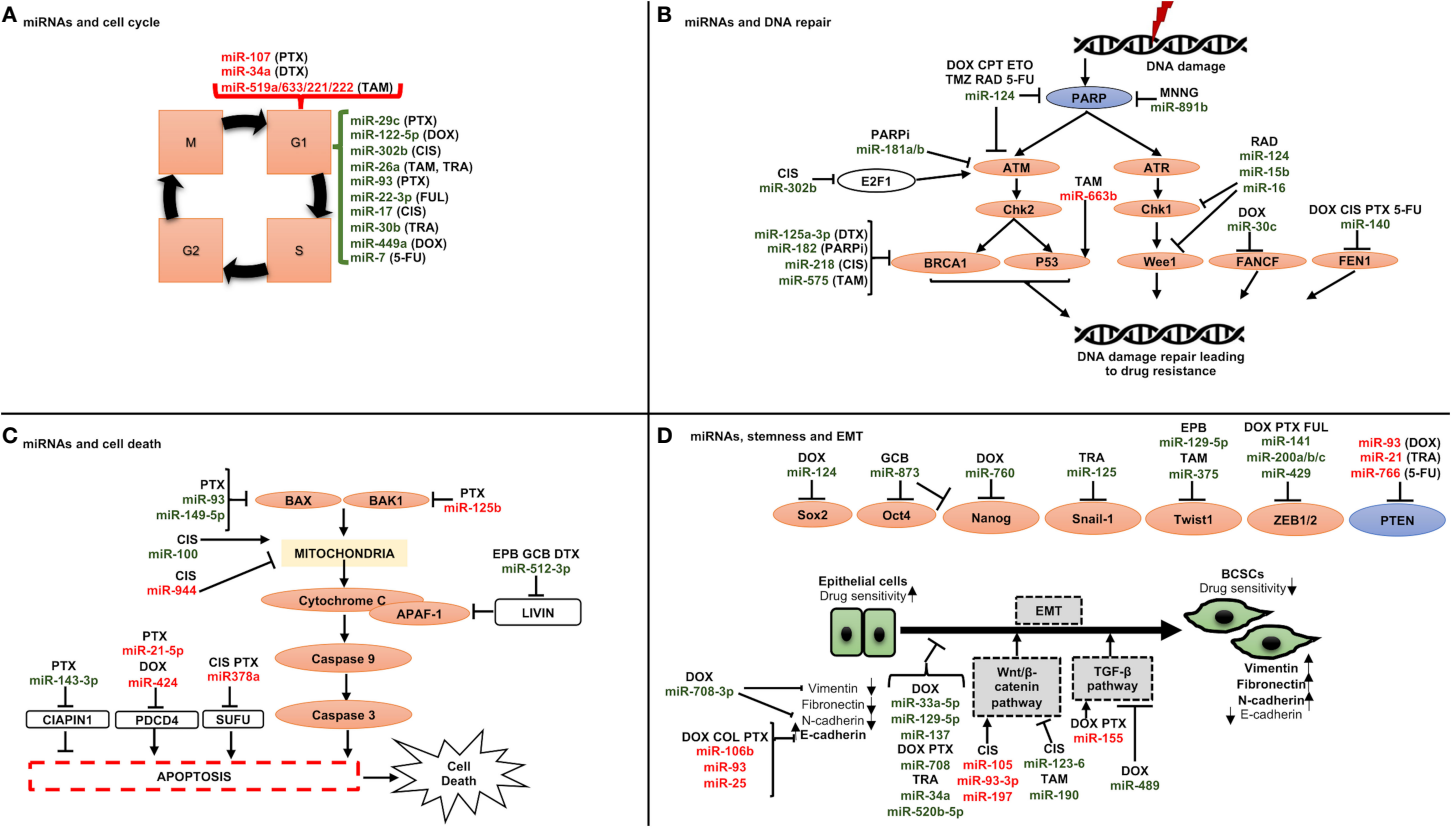
Schematic representation of miRNAs involved in drug resistance through regulating **(A)** cell cycle, **(B)** DNA repair checkpoints, **(C)** cell death, and **(D)** stemness and epithelial to mesenchymal transition. A line with a perpendicular line at the end designates inhibition and arrows designate activation. miRNAs increasing drug resistance are shown in red, and miRNAs increasing drug sensitivity are shown in green. CIS, cisplatin; DOX, doxorubicin; DTX, docetaxel; FUL, fulvestrant; PTX, paclitaxel; TAM, tamoxifen; TRA, trastuzumab; 5-FU, 5-fuorouracil; CPT, camptothecin; ETO, etoposide; MNNG, N-methylN′-nitro-N-nitrosoguanidine; PARPi, PARP inhibitors; RAD, radiation; TMZ, temozolomide; EPB, epirubicin; GCB, gemcitabine.

The role of miRNAs in BC chemoresistance has been attributed to some of the following molecular mechanisms (**Figure 3**):

♦ *miRNAs and cell cycle:* Cell cycle deregulation is an established hallmark of cancer, and it has been linked to both drug resistance and poor prognosis when it is aberrantly activated. Various miRNAs have been reported to target genes linked to cell cycle regulation, resulting in either drug sensitivity or resistance such as miR-93, involved in G1/S phase arrest, was reported to be downregulated in paclitaxel-resistant BC samples compared to responder patients (**Figure 3A**) (151). Direct targets of this miRNA were discovered to include CCND1 and the E2F transcription factor 1 (E2F1), which upon downregulation resulted in cell cycle arrest in G1 phase and increased apoptosis *via* inhibiting AKT phosphorylation (p-AKT), and BCL-2 expression, and increasing the expression levels of BCL-2-associated X, apoptosis regulator (BAX), which could increase the paclitaxel sensitivity.
♦ *miRNAs and DNA repair machinery:* As mentioned above, most chemotherapy drugs used today to treat breast cancer cause either direct or indirect DNA damage. To counteract DNA damage, however, CSCs activate DDR pathways, explaining why chemotherapy that destroys DNA could result in drug resistance (**Figure 3B**). One such DDR pathway involves BRCA1 which is engaged in different cellular processes that maintain genomic stability like DNA damage repair, DNA damage-induced cell cycle checkpoint activation, chromatin remodelling, protein ubiquitination, transcriptional control, and cell death [43]. Drug sensitivity is thus impacted by its miRNA influenced control, for instance miR-182 inhibits BRCA1 expression to induce drug sensitization. Furthermore, it has been shown that overexpressing miR-182 makes BC cells more susceptible to PARPi (PADP-ribose polymerase 1 inhibitor). In contrast, miR-182 suppression raises BRCA1 levels and results in PARPi resistance (152).
♦ *miRNAs and cell death:* The interests of investigators are growing in drug-miRNA combination anticancer therapy since miRNAs can influence cell death (**Figure 3C**). Examples include miR-125b, which confers paclitaxel resistance by inhibiting the expression of BAK1 (BAX and/or BCL-2 Antagonist/Killer 1), which causes the release of cytochrome C from mitochondria to the cytoplasm, where it binds Apoptotic peptidase Activating Factor 1 (APAF-1) and triggers caspase activation (76). Similar findings were also made from miR-149-5p, whose overexpression was shown to boost BAX expression (153), and from miR-663b, which imparts tamoxifen resistance by indirectly upregulating BAX (154).
♦ *miRNAs, CSCs and epithelial to mesenchymal transition (EMT):* Breast cancer stem cells (BCSCs) are a small population of cells with a high ability for tumorigenesis and are involved in therapy resistance (155). The modulation of BCSCs’ phenotype is mediated by several molecular mechanisms, the most significant of which is EMT. This process takes place as cancer develops, and it involves a decrease in the expression of molecules associated with epithelial growth, such as E-cadherin, and a rise in molecules associated with mesenchymal development, such as N-cadherin, vimentin (VIM), and fibronectin (FN1) (156). Thus, the cells become more capable of invasion and migration (155) and can nest in various tissues where they can multiply and create new tumors through a process called metastasis (155). In this context, miRNAs play a significant role in controlling stemness and EMT by targeting a few genes implicated in these two pathways (**Figure 3D**). Among those engaged in the control of EMT, the miR-200 family has received the greatest research attention. Five different miRNAs make up this family: miR-141, miR200a, miR200b, miR200c, and miR-429 (157), which can inhibit the expression of ZEB1 and ZEB2 (Zinc finger E-Box-binding homeobox genes) (157). As a result, it has been demonstrated that overexpressing miR-200 in many cancer cell lines can reverse EMT (158). Another factor contributing to stemness in BC is the Wnt/-catenin signaling pathway. It has been shown that several miRNAs, including miR-105 and miR-93-3p, regulate this pathway. The Wnt/-catenin signaling pathway suppressor Secreted Frizzled Related Protein 1 (SPFR1) is the target of those miRNAs. This led Li et al. to show that those miRNAs encourage cisplatin resistance (159).

Four circulating miRNA patterns linked to pCR were recently identified using profiling of circulating miRNA (ct miRNA detected in plasma) to categorize NAC responders (from non-responders) in Her2+ patients (160). These results demonstrate the potential of miRNA signatures as prognostic and predictive biomarkers that could individualize breast cancer treatments and enhance patient sampling techniques for current therapies, including traditional cytotoxic chemotherapies. The following is a discussion of a few of them:

♦ *miR-638* – miR-638 was shown to be downregulated in cases with BC chemoresistance in a microarray analysis (161). A minimal patient-derived xenograft (MiniPDXTM) was also developed by the researchers to assess the chemosensitivity of various drugs. The results of this study demonstrated that in patients, who received 5-FU, miR-638 levels were relatively low in the 5-FU-resistant group compared to the 5-FU-sensitive group. So, according to the MiniPDX™ model, MDA-MB-231 BC cells overexpressing miR-638 were more susceptible to 5-FU treatment *in vivo*.
♦ *miR-17/20* – The serine-threonine kinase Akt1 has been linked to the regulation of cellular homeostasis, proliferation, and growth as well as hyperactivation in human malignancies (162). It is known that the miR-17/20 cluster blocks the proliferation of breast cancer cells by causing the G1/S cell cycle arrest by anchoring to the 3’UTR of cyclin D1 (163). Yu et al., 2014 demonstrated a unique mechanism by which miR17/20 controls p53 and Akt, which further control breast cancer cell apoptosis (163). Additionally, they have demonstrated that miR-17/20 overexpression, *via* Akt1, makes MCF-7 cells more susceptible to apoptosis brought on by either doxorubicin or UV irradiation. In brief, the apoptosis-inducing miR-17/20 increases Akt breakdown by upregulating p53 expression.
♦ *miR*-*342* – The mesenchymal stem cell-derived exosome (MSCs-Exo) carrying microRNAs has been proven to regulate tumor biological activities (164, 165). Yu et al., 2022 demonstrated the regulatory function of miR-342-3p in MSCs-Exo on the BC (166). They revealed considerably decreased levels of miR-342-3p in patients with metastatic illnesses (166). Additionally, miR-342-3p was found to target the Inhibitor of Differentiation 4 (ID4) and operate as a possible tumor suppressor by preventing the metastasis and chemoresistance of breast cancer cells (166).
♦ *miR*-*484* –By controlling cyclin E-CDK2 signaling, cytosine deaminase (CDA), a crucial chemoresistance axis, inhibits the advancement of the cell cycle (167). In a breast cancer model that is resistant to gemcitabine, miR-484 controls the CDA (167). CDA expression was found to be downregulated and inversely linked with miR-484 expression in clinical samples of BC (167). Additionally, in the same cohort, greater expression of CDA was linked to extended disease-free survival. Collectively, the findings of Ye et al., 2015 established that miR-484-modulated CDA promotes chemoresistance while inhibiting cell proliferation in breast cancer, highlighting the pathophysiological exchange that arises because of chemoresistance in this cancerous condition (167).
♦ *miR-451* – Gu et al. (2016) looked at the possible use of miR-451, which is prevalent in the serum of BC patients, to forecast NAC resistance (168). Here, qRT-PCR was used to determine the expression levels of miR-451 in the MCF-7 BC cell line, the docetaxel-resistant MCF-7 BC cell line (MCF-7/DTX), the epirubicin-resistant MCF-7 BC cell line (MCF-7/EPB), normal controls, NAC-sensitive group, and NAC-resistant group. The findings of this investigation confirmed the hypothesis that miR-451 expression was differentially expressed between NAC-sensitive and -resistant BC patients. Additionally, the research team noticed that miR-451 expression was much lower in the MCF-7/EPB and MCF-7/DTX cell lines than it was in the MCF-7 cell lines, indicating that miR-451 may be functionally crucial in predicting NAC resistance in breast cancer patients.
♦ *miR-222 and miR-29a –* In a 2013 study by Zhong et al., it was discovered that the changed expression pattern of miR-222 and miR-29a contributed to the development of DTX and adriamycin (ADR) resistance in breast cancer MCF-7 cells (128). The research team found that targeting Phosphatase and TENsin homolog (PTEN) with miR-222 and miR-29a mimics and inhibitors partially altered the treatment resistance of breast cancer cells.
♦ *miR-141 –* About 50% of patients develop resistance to the chemotherapeutic medication docetaxel used to treat BC. The role of miR-141 expression in BC cells with acquired docetaxel resistance was studied by Yao et al., 2015 (122). Docetaxel-resistant cells (MCF-7/DTX and MDA-MB-231/DTX, respectively) were more responsive to the drug when miR-141 was inhibited, but docetaxel-sensitive cells were more resistant when miR-141 was overexpressed (MCF-7 and MDA-MB-231, respectively). This research team showed that miR-141 operates on genes required for drug-induced apoptosis, leaving the cells drug resistant, through direct interaction with eukaryotic translation initiation factor 4E (EIF4E).
♦ *miR-140-5p –* In a training set, conducted by Di et al., 2019, starting from 51 circulating (ct)-miRNAs linked with pCR, four signatures were validated in the testing set: lapatinib at T0 and T1 [AUC 0.86; 95% confidence interval (CI), 0.73–0.98 and 0.71 (0.55–0.86)], respectively; trastuzumab at T1 (0.81; 0.70–0.92); lapatinib + trastuzumab at T1 (0.67; 0.51–0.83) (160). Although the levels of ct-miR-140-5p, which is a component of the trastuzumab signature, were linked to EFS (HR 0.43; 95% CI, 0.22-0.84), ct-miRNA signatures could not predict event-free survival (EFS). Patients with and without pCR after neoadjuvant lapatinib- and/or trastuzumab-based therapy can be distinguished by ct-miRNAs. To help to de-escalate treatment plans, ct-miRNAs at week two may be useful in identifying individuals who respond to trastuzumab and preventing needless combinations with other anti-HER2 medications.
♦ *miR-34a –* A link between enhanced miR-34a expression and docetaxel resistance has also been established (80). Kastl et al., 2012 confirmed that B-cell leukemia/lymphoma 2 protein (BCL-2) and cyclin D1 protein (CCND1), both of which are targeted by miR-34a, were shown to be expressed at lower levels in docetaxel-resistant cells (80). It was found that overexpressing miR-34a resulted in resistance in MCF-7 docetaxel-sensitive cells, but miR-34a inhibition improved responsiveness to docetaxel in MCF-7 docetaxel-resistant cells. To propose a prospective therapeutic target for the treatment of docetaxel-resistant breast cancer, this work described a pathway of acquired docetaxel resistance in these cells, presumably involving direct interactions with BCL-2 and CCND1.
♦ *miR-23, 24 and 27* – Recent research has shown that the extracted exosomes (D/exo) from the docetaxel-resistant breast cancer cells MCF-7 (MCF-7/Doc) were linked to the genetic cargo’s contribution to resistance transmission (169, 170). The significance of D/exo during exposure to DRβ-H (d Rhamnose -hederin), an active ingredient obtained from the traditional Chinese medicine plant *Clematis ganpiniana*, was discovered by Chen et al., 2018, in MCF-7/DTX cells (171). Herein, the investigators have found that DRβ-H could reduce the expression of a few of the most common miRNAs (*miR-23a, miR-24*, and *miR-27a*) transported by D/exo. Target gene prediction and pathway analysis showed the relevance of these selected miRNAs in pathways related to disease relapse.
♦ *miR-200* –The miR-200 family of microRNAs have recently been revealed to be dysregulated in a variety of malignancies, and it has been shown that this family of miRNAs is crucial for tumor development, maintenance, tumor metastasis, and chemotherapy tolerance (172). MiR-200s are currently recognized as master EMT regulators, inhibiting cancer invasion and metastasis by focusing on a number of key inducers of the EMT, such as ZEB1, ZEB2, and SLUG (172). By playing critical and pleiotropic roles in malignancies, miR-200s are promising targets for cancer therapy. However, a recent study revealed that miR-200s play a role in breast cancer metastasis promotion, hence cautious evaluation should be done prior to treatment modalities using miR-200s as therapeutic targets (172).

## 8 miRNAs in neoadjuvant chemotherapies: predicting response

As already said, breast oncology research has advanced recently to realize that treating patients with chemotherapy in the neoadjuvant setting is both rational and beneficial (173, 174). Although conventional clinicopathological traits have been shown to correlate with response to NAC (33), it is still difficult for oncologists to identify patients who are likely to experience such reactions since success rates are frequently unpredictable. The latest research has linked miRNA expression profiles with breast cancer patients’ responses to NAC therapy. For example, Xing et al., 2021 found that decreased expression of miR-638 and miR-451a and elevated expression of miR-200c-3p, miR-23a-3p and miR-214-3p correlated to chemoresistance (Miller–Payne grade 1) (175). In their analysis of 114 breast cancer patients participating in the Clinical Trials Ireland All-Ireland Cooperative Oncology Research Group (CTRIAL-IE ICORG) 10/11 prospective, multicenter translational trial, McGuire et al., 2020 emphasize the innate value of miR-21 expression as a factor associated to response to conventional NAC (176). Further, a study conducted by Liu et al., 2019 supported the findings of the CTRIAL-IE ICORG 10/11 trial by showing decreased miR-21 expression levels in responders (*vs* non-responders) following cycle 2 of NAC (177). Di Cosimo et al., 2020 described the clinical utility of venous sampling for miR-140a-5p, miR-148a-3p, and miR-374a-5p, and their predictive value in determining response to subsequent neoadjuvant therapy, with an enhanced combined predictive capability of 54% in determining pCR to trastuzumab in HER2+ illness, compared with 0% in cases of poor expression (178). In their series of 435 patients with either early-stage HER2+ or TNBC illness, Stevic et al. (2018) explained how the overexpression of miR-199a in patient plasma was indicative of pCR to NAC in the GeparSixto study (179). MiR-34a expression levels were shown to accurately distinguish between responders and non-responders in 39 patients receiving treatment for locally advanced breast cancer according to promising findings from Kassem et al., 2019 (area under the curve (AUC): 0.995, sensitivity: 97.4%, specificity: 100%) (180). In patients who successfully achieved a pCR to NAC, Garcia et al., 2019 showed lower miR-145-5p expression levels (AUC: 0.790, p = 0.003) (181). **Table 4** shows systematic trials examining the function of miRNAs in determining how patients would respond to neoadjuvant therapy and lists the miRNAs that are important in this setting (160, 176, 178, 179, 183, 184, 186–190). Using miRNA expression profiles to assess response to adjuvant chemotherapy is substantially more difficult. It is quite challenging to measure whether medication improved oncological outcomes for patients who were most likely to succumb to recurrence, estimate the timing of miRNA sampling, and analyze treatment response rates in a crude way. Therefore, it is not surprising that most research evaluates miRNA expression patterns using metrics that indicate response to NAC rather than adjuvant chemotherapy (e.g., RECIST, Miller-Payne grade, Sataloff score, etc.).

**Table 4:** Table illustrating prospective trials evaluating the role of miRNAs in predicting response to neoadjuvant therapies.

Author	Year	Trial Phase	Trial Number/Link	N	Treatment Arms	Findings	References
Jung	2012	Prospective (II)	N.A.	72	5-FU, EC and trastuzumab	Lower miR-210 expression levels predicted pCR in HER2+ cancers.	(182)
Muller	2014	Prospective phase II Geparquinto Trial	NCT:00567554	127	NAC with trastuzumab or lapatinib	Increased miR-21, miR-210, and miR-373 in patient’s serum following treatment with NAC correlated to response to treatment.	(183)
Xue	2016	Prospective phase II clinical trial	N.A.	50	Carboplatin and Paclitaxel	Increased miR-621 expression profiles predicted pCR to NAC	(184)
Al-Khanbashi	2016	Prospective (II)	N.A.	36	DXR, cyclophosphamide and DTX	Serum miR-451 expression levels decreased during NAC in clinical responders.	(185)
Stevic	2018	Prospective phase II clinical trial GeparSixto Trial	NCT:01426880	211	Docetaxel or Paclitaxel +/− Carboplatin	Aberrant miR-199a expression correlates to pCR following neoadjuvant therapies	(179)
Zhu	2018	Prospective phase II clinical trial	NCT:02041338	24	Epirubicin and Docetaxel	After the second cycle of NAC, reduced miR-34a expression was correlated with patients who did not respond to treatment	(186)
Kahraman	2018	Prospective, case–control study (MODE-B study)	N.A.	42	Carboplatin and Paclitaxel	Identification of 74 miRNAs which predicted pCR based on changes in expression profiles pre- and post-NAC.	(187)
Di Cosimo	2019	NeoALLTO Phase III RCT	NCT:00553358	455	Neoadjuvant lapatinib, trastuzumab, or combined lapatinib and trastuzumab	Increased circulating plasma levels of miR-140a-5p, miR-148a-3p and 374a-5p were associated with pCR and miR-140a-5p predicted enhanced EFS	(160)
Lindholm	2019	Randomised, phase II clinical trial	NCT:00773695	132	FEC-T or FEC-P, +/− Bevacizumab	Hierarchical clustering of 627 miRNAs with response at 12 and 25 weeks to neoadjuvant treatment with NAC or NAC combined with Bevacizumab; of these, 217 had differential expression profiles (71 upregulated and 146 downregulated) between responders and non-responders.	(188)
Rodriguez-Martinez	2019	Prospective clinical trial	N.A.	53	AC	Exosomal expression of miR-21 correlated in a stepwise fashion with patients achieving a CR having significantly reduced miR-21 *vs*. patients with PR and SD, respectively	(189)
Di Cosimo	2020	NeoALLTO Phase III RCT	NCT:00553358	455	Neoadjuvant lapatinib, trastuzumab, or combined lapatinib and trastuzumab	After 2 weeks of neoadjuvant treatment, increased expression of miR-15a-5p, miR-140-3p, miR-320a, miR-320b, miR-363-3p, miR-378a-3p, miR-486-5p and miR-660-5p and decreased miR-30d-5p correlated with pCR to lapatinib. At 2 weeks of therapy, increased expression of miR-26a-5p and miR-374b-5p correlated with pCR to trastuzumab. Increased let-7g-5p and miR-191-5p and reduced miR-195-5p correlated with pCR to combined trastuzumab and lapatinib.	(178)
McGuire	2020	Prospective phase II clinical trial [CTRIAL-IE ICORG] 10/11	NCT:00553358	114	Various NAC regimens	Responders had reduced miR-21 and miR-195 *vs*. non-responders in all breast cancer subtypes. MiR-21 independent predicted response (OR 0.538, 95% CI 0.308–0.943). In luminal cancers, reduced expression of miR-145 and miR-21 correlated with response to NAC.	(176)
Zhang	2020	Prospective phase II trials	SHPD001 NCT:02199418 and SHPH02	65	Paclitaxel, Cisplatin and trastuzumab	Low miR-222-3p expression was predictive of achieving pCR (OR: 0.258, 95% confidence interval: 0.070–0.958, *p* = 0.043) and favourable DFS and survival	(190)

N.A., Trial number/link not available; N, number; HER2-+ human epidermal growth factor receptor-2 positive NAC, neoadjuvant chemotherapy; pCR, pathological complete response; TNBC, triple-negative breast cancer; EFS, event-free survival; HER2-, human epidermal growth factor receptor-2 negative; FEC-T, 5-fluorouracil; epirubicin; and cyclophosphamide followed by docetaxel; FEC-P, 5-fluorouracil; epirubicin; and cyclophosphamide followed by paclitaxel; AC, doxorubicin and cyclophosphamide; CR, complete response; PR, partial response; SD, stable disease; OR, odds ratio; DFS, disease-free survival; NCT, national clinical trial identifier; TAN, tumor-associated normal; DTX, docetaxel; DXR, doxorubicin.

## 9 MicroRNAs for therapeutic use in breast cancer

**Figure 4:**
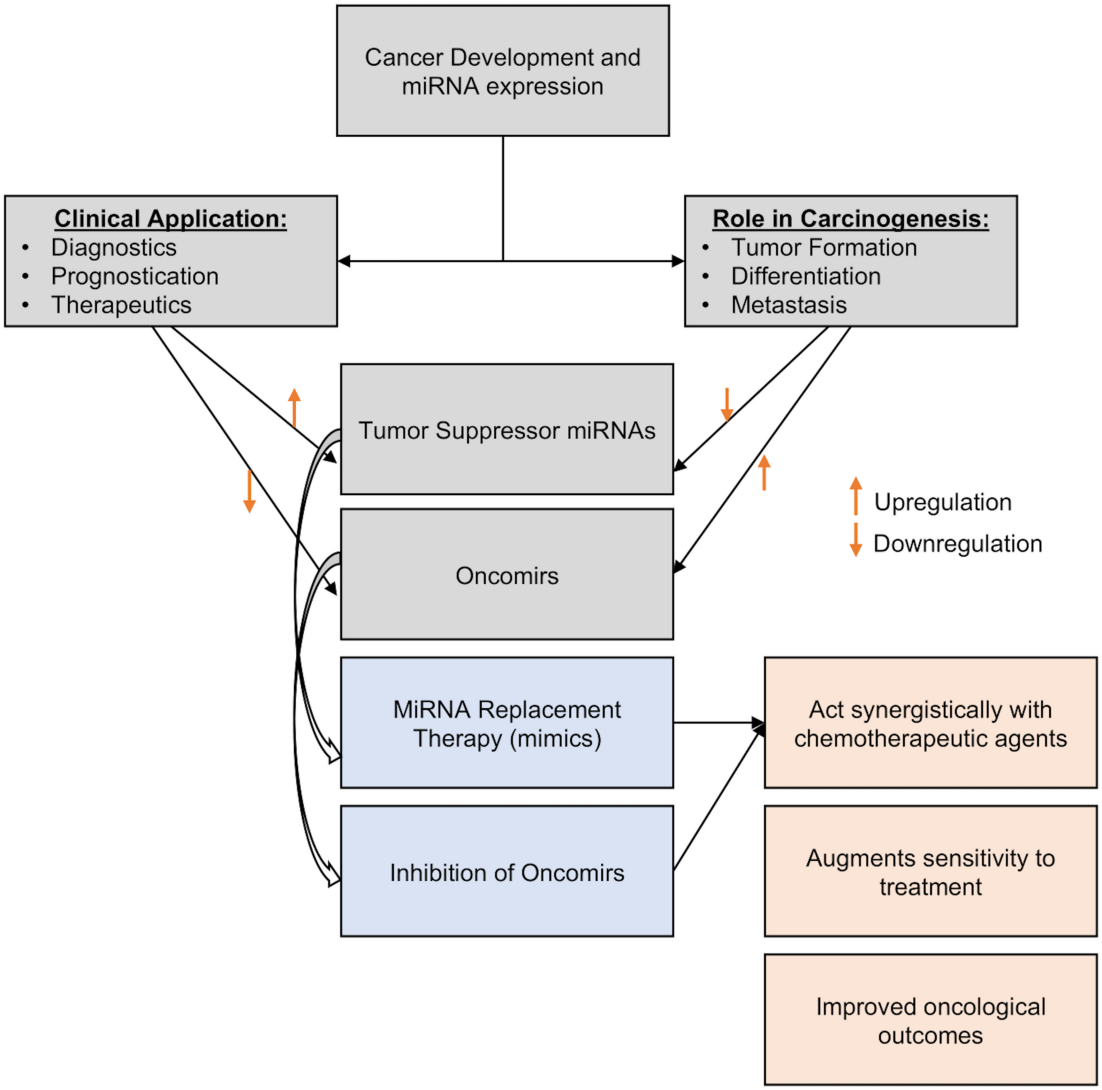
Figure showing how miRNA expression profiles can be altered for cancer treatment.

The use of miRNAs for the development of novel treatment approaches has been made easier by the current molecular technology. These entail the administration of carefully chosen miRNAs into the tumor microenvironment for therapeutic purposes or to improve the efficacy of currently available therapeutic modalities employed in standard clinical practice, such as systemic chemotherapy (143, 191). miRNAs can act as tumor suppressors or oncomirs, so there are two possible methods for using them as therapeutics (1): miRNA replacement therapy, which involves inducing and overexpressing specific miRNA to reduce oncogenesis or increasing sensitivity to systemic treatment, or (2) oncomir inhibition, which involves lowering targeted miRNA expression characteristics (i.e., miRNA silencing) by incorporating inhibitory miRNA to lessen the translation of the target miRNA (**Figure 4**).

♦ *miRNA Replacement Therapy* – By inhibiting oncogenes and the genes that regulate cell proliferation and death, tumor suppressor miRNAs can prevent the development of cancer (192). MiRNA replacement treatment includes reintroducing tumor-suppressing miRNA (or mimics) into the tumor microenvironment in order to inhibit tumor growth and restrain the spread of malignancy (193). They might be delivered into the cytoplasm of cancer cells through a variety of transporters, such as chemicals, electroporation, and modelling of the endogenous miRNA (192). Park et al., 2014 discussed the possible significance of overexpression of miR-34a in MCF7 cells in reducing cancer stem cell characteristics and increasing sensitivity to doxorubicin treatment by specifically targeting NOTCH1 (194). In studies involving animals and MDA-MB-231 and MDA-MB-549 chemoresistant breast cancer cell lines, Yu et al., 2007 and 2010 and Cochrane et al., 2009 show the value of gradually introducing and increasing the expression levels of let-7a, miR-30, and miR-200c to minimize oncogenesis and increase therapeutic index (195–197). Additionally, Kalinowski et al. (2014) analyzed how miR-7 replacement therapy can improve the efficacy of the traditional breast cancer chemotherapy currently being used to treat breast cancer (198).
♦ *Oncomir Inhibition* – Generally, oncomirs are elevated in the cancer (191). Anti-miRNA oligonucleotides, targeted miRNA silencing agents (antagomirs), and locked nucleic acids (LNA) can all be used to limit the action of oncogenic miRNAs (199). In various pre-clinical studies, such inhibitor mechanisms have been shown to increase the sensitivity of breast cancer cells to chemotherapeutic agents: For example, in MCF-7/ADR cell lines, miR-3609 was successfully transfected to increase the tumor cells’ susceptibility to adriamycin-based chemotherapy (200). Similarly, Lin et al., 2021 successfully enhanced cell sensitivity to chemotherapy in 65 BC patients by inducing miR-133 into cisplatin-resistant TNBC cells from these patients (201). Li et al., 2021 also successfully overcome paclitaxel resistance in previously resistant breast cancer cells by transfecting miR-155-5p into tumor cells (85). Finally, Mei et al., 2010 report that downregulating miR-21 increased the susceptibility of MCF-7 BC cell lines to docetaxel treatment (202).

### 9.1 miRNA delivery strategies used for cancer therapy

**Figure 5:**
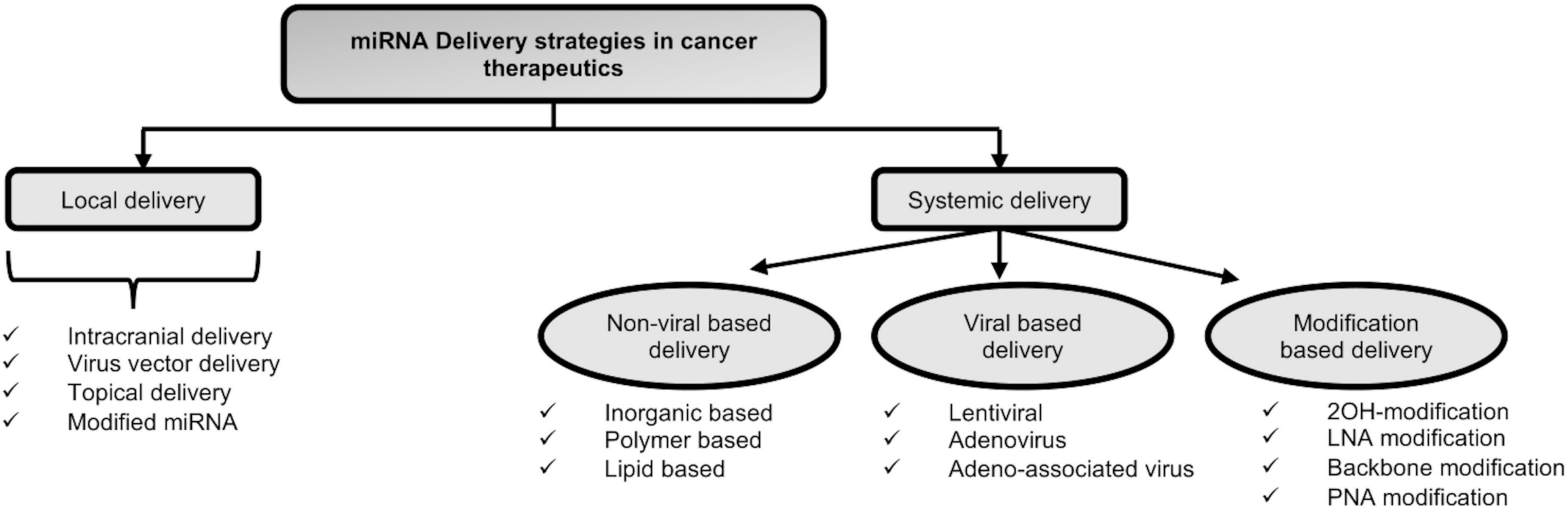
Types of microRNA delivery techniques employed in cancer therapy.

miRNAs can be introduced therapeutically into cancer cells through a variety of methods. These approaches are typically divided into two categories of local and systemic delivery, which are thoroughly discussed below and in **Figure 5**:

***Local delivery of miRNAs:*** Target gene suppression with less toxicity may result from the local delivery of miRNAs as opposed to the systemic delivery of miRNAs. According to Møller et al., 2013, the aforementioned strategy has been examined mainly for primary tumors including melanoma, breast, and cervical cancers (203). Recently, different local delivery techniques, such as the direct injection of miRNA vectors into the tumor site and the nanoparticles (NP) formulation with surface modifications, have been devised. For instance, glioblastoma multiform was treated using the intracranial miRNA delivery approach (203). In a study by Trang et al., 2010, let-7 was introduced into non-small-cell lung cancer using viral vectors, which inhibited the growth of KRAS-dependent tumors (204). The topical distribution approach is an additional technique for treating skin conditions. The target region is more accessible with fewer adverse effects when topical administration is used (205). Moreover, the local delivery system makes the use of modified miRNAs. For instance, astro-cyte elevated gene-1 (AEG-1) was the target of intratumoral miR-375 mimics in cholesterol-conjugated 2′-O-methyl modified form, which significantly suppressed tumor growth in *in-vivo* models of hepatoma xenograft (206).

However, as the local delivery system employs direct injection or local application of miRNAs with or without carriers, it cannot be recommended as a good strategy for treating late-stage metastatic disease. Therefore, developing a systemic delivery strategy is essential to provide efficient miRNA cancer therapy.

***Systemic delivery of miRNAs:*** The systemic miRNA delivery technique represents a significant advancement in the effort to increase the effectiveness of cancer therapy and get over the drawback of miRNA delivery *in vivo*. Different systemic delivery strategies have been devised up until this point. A few of them are covered below:

♦ *Modification-based delivery* - Systemic distribution of miRNAs into tumor cells was mostly accomplished through chemical alterations (207). The altered oligonucleotides show a stronger propensity for the target molecule. However, there are some restrictions in this regard. For instance, a targeting moiety is necessary for the intracellular uptake of modified miRNAs. Additionally, there might be a sign of short half-lives and uneven biodistribution because of quick renal and hepatic clearance. The stability of miRNA modulators and their resistance to nuclease degradation inside the blood circulation may both be improved by increasing the systemic delivery effect with various chemical modifications (208). These changes lessen the off-target effects of miRNAs and aid to overcome immune responses and low miRNA stability (207). The typical chemical modifications are 2′‐OH group modification, LNA modifications, passenger strand modifications, Phosphorothioate modification and peptide nucleic acid (PNA) modifications.
♦ *Viral delivery of miRNAs* – miRNAs can be transmitted by being encoded in several types of vectors, including viral and non-viral vectors. In this regard, viral delivery is an advantageous strategy. One of its benefits include low off-target rate, resulting from given miRNAs being translated by tumor cells. Lentiviruses, adenoviruses, and adenoassociated viruses (AAVs) are among the viruses that have been identified as delivery vectors for miRNAs. As a result, targeting components were added to the viral capsid to strengthen the affinity between viral vectors and cancer-specific receptors, allowing for better transportation into tumors (209). However, due to the immunological reaction they cause and the difficulty of scaling up the production process in comparison to nonviral delivery systems, there are still some significant challenges to overcome. Additionally, the potential for a virus with replication competence may raise the risk of the pathogenic condition. For instance, some retroviruses can cause the start of a CNS illness as a result of their active reproduction (210).
♦ *Non-viral delivery of miRNAs* – The use of non-viral vectors is a beneficial strategy for miRNA delivery. In this approach, site-specific delivery, system optimization, or polyethylene glycol (PEG) molecule augmentation could be employed to achieve targeted ligands or lengthen circulation times. Additionally, nanocarriers are produced in a secure and straightforward manner, and they are distinguished by their affordability, minimal immunogenicity, and adaptability. Non-viral delivery vectors can be divided into three primary categories: inorganic materials, lipid-based carriers, and polymeric carriers (211, 212).
However, non-viral-based approaches to miRNA delivery have their own shortcomings such as lower loading efficiency, lack of cargo protection, lower endosomal escape, nonspecific interaction with target cells and nucleic acids, etc (193).

## 10 Discussion

Considering that drug resistance continues to be a major obstacle in the clinical context, causing relapse and metastatic spread in many cancer types, novel treatment approaches are of the utmost importance. The discovery of miRNAs has provided a novel perspective on the molecular processes behind cancer, increasing the possibility of creating novel and more potent therapeutic approaches. This review is centered on new findings pertaining to the significance of miRNAs in breast cancer chemoresistance. miRNAs regulate numerous signaling pathways and regulatory networks, therefore even little changes in miRNA expression can have a big impact on the development and the progression of the disease. Targeting miRNAs—either reducing or enhancing their expression—seems promising to develop novel, more effective, and customized treatments, boost therapeutic efficacy, and predict patient response to various treatments. However, to fully explain all the miRNAs that are altered in tumors based on profiling data would be beyond the scope of this review. Numerous organizations are exploring the use of microRNAs as potential therapeutics. *In vivo* and translational investigations are currently the focus of increased research. Evidence exists that points to miRNAs as possible therapeutic agents, particularly when used in conjunction with anti-cancer chemotherapeutics. This could take the form of mimics that support miRNA function and expression or antagonists that block miRNA expression. By affecting the expression of endogenous microRNAs in cancer cells, miRNA mimics or anti-miRNAs can potentially change chemotherapy’s efficacy. Two clinical investigations have shown the potential therapeutic impact of miRNAs in the future. Among these is a phase 2a clinical trial with Miravirsen in 26 patients who had chronic hepatitis C virus (HCV) genotype 1 infection. Miravirsen is a nucleic acid–modified DNA phosphorothioate antisense oligonucleotide that encases mature miR-122 in a heteroduplex and inhibits its function. No side effects related to the experiment have been reported yet (213). Another phase 1 clinical trial including individuals with liver cancer or metastatic cancer with liver problems is MRX34 (a mimic of the tumor suppressor miR-34). Healthy volunteers and patients with advanced or metastatic liver cancer (hepatocellular carcinoma) are being tested for the safety and effectiveness of MRX34 in this study (214). Future possibilities for these novel medicines are encouraging given the encouraging preliminary findings from both trials.

**Figure 6:**
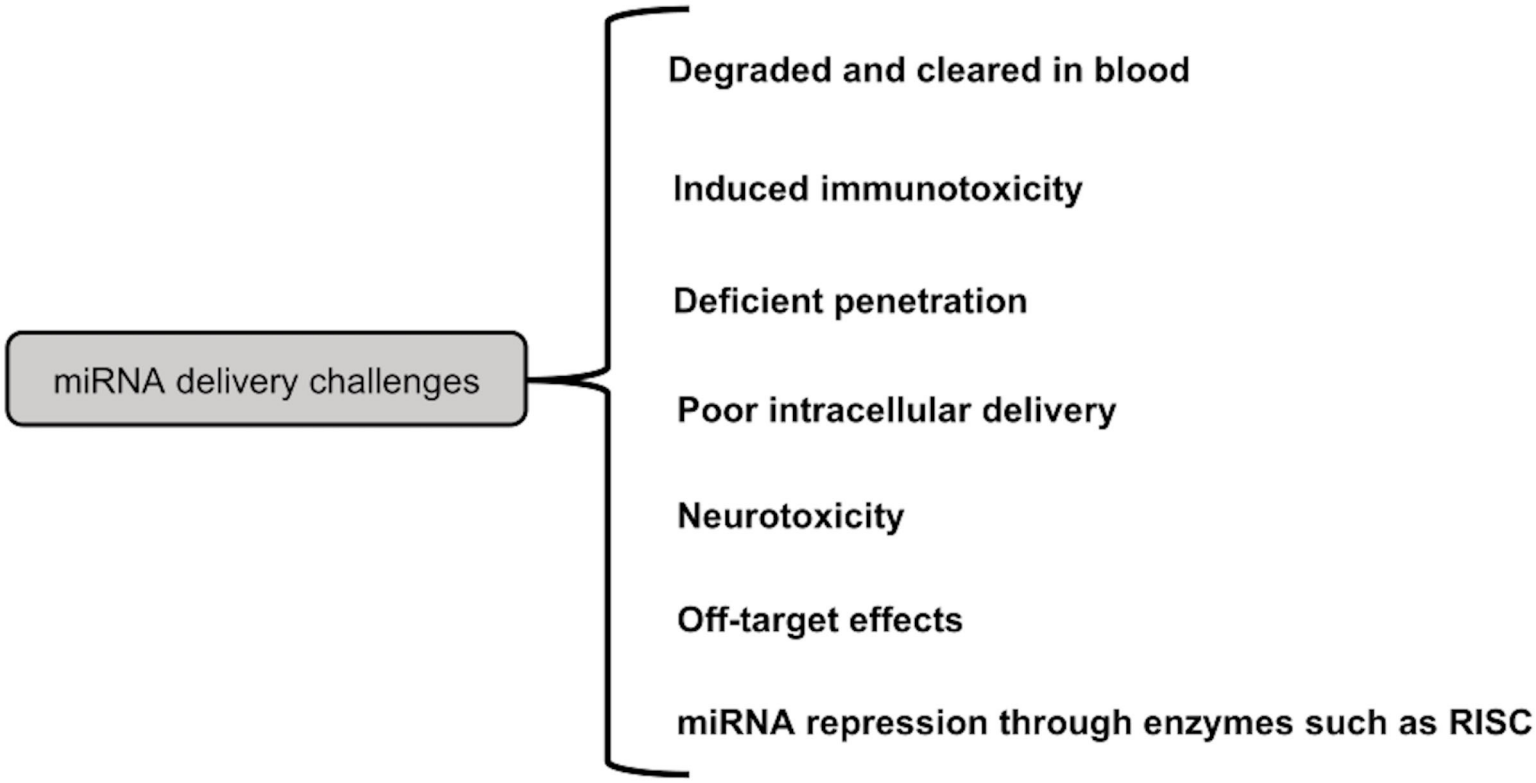
Challenges in miRNA delivery.

**Challenges in the field of miRNA therapy-**As mentioned above, the miRNAs can be delivered by either local or systemic approaches. It might not be a suitable strategy, for advanced cancer. However, miRNA cancer therapy works well with systemic delivery. **Figure 6** summarizes the various constraints to miRNA delivery. For instance, poor miRNA penetration is caused by the leaky nature of aberrant tumor vasculature (215). The rapid cleavage of naked miRNAs by serum nucleases of the RNase A type poses another challenge (216). Additionally, there is a rapid renal clearance, notably for naked miRNA (217). When utilizing big NPs (>100 nm), reticuloendothelial system (RES) clearance would rise in the liver, spleen, lung, and bone marrow, leading to nonspecific absorption by innate immune cells such monocytes and macrophages (218).

Additionally, the systemic miRNA distribution triggered the innate immune system, as with other nucleic acid types, which resulted in undesired toxicities. Immune system activation includes the release of inflammatory cytokines and Type I IFNs *via* Toll-like receptors (TLRs) (219). Anti-inflammatory miRNA treatment, however, may prevent the activation of inflammatory pathways (220). On the other hand, some miRNAs work through TLRs to trigger neurodegeneration. For instance, Lehmann et al. (2012) demonstrated that miRNA let7b can cause neurotoxicity by activating TLR7 signaling in neurons (221). Therefore, a significant issue for miRNA systemic cancer therapy is the incidence of miRNA-related neurotoxicity. Additionally, increased miRNA uptake in cancer cells is a problem, and methods to address this issue include increasing endosomal escape and releasing miRNA payloads into the cytoplasm.

Off-target effects brought on by the miRNA mode of action are yet another challenge for miRNA delivery systems. These compounds may have undesirable side effects since they may bind to the 3′-UTR of a number of genes and decrease their expression (222). A developed method to lessen these adverse effects is the use of multifunctional co-delivering systems (223). Furthermore, it was demonstrated that under specific circumstances, such as hypoxia, the activity of miRNA processing enzymes such RISC reduces, which lowers the expression of tumor suppressor miRNAs (224). De Carvalho Vicentini et al. (2013) suggest that altering the expression or activity of these enzymes is another method for suppressing miRNA (225).

## 11 Conclusion

The discovery, development, and enhancement of miRNAs as potential medicines for the treatment of breast cancer patients have received significant funding, yet this branch of translational research is still in its infancy. Numerous attempts have been made to tailor cancer therapies using miRNA, but little progress has been made in improving clinic-oncological outcomes using miRNA targeting. miRNA therapies are now facing several developmental obstacles. This study is constrained by the fact that most of the research done thus far provides information about *in-vitro* trials, with very few studies coming from sources other than animal or breast cancer cell lines. Clinical trials assessing the clinical effectiveness, risk profiles, and premium benefit are necessary for addition to the generally accepted scientific technique to support the initial findings of these recent investigations. An in-depth discussion of how clinical trial research has transformed BC patient care over the last four decades is provided in the current review. This research has produced novel, individualized therapeutic approaches, minimally invasive surgical techniques for the breast and axilla, and improved clinico-oncological results for patients who might otherwise have died from their disease in earlier times. The personalization of BC patient care appears to be closer than ever thanks to ongoing trials evaluating cutting-edge targeted therapies like immune checkpoint modulation (39, 226) and the use of poly(adenosine diphosphate-ribose) polymerase inhibitors (or PARP inhibitors) in the treatment of early-stage breast cancer in BRCA mutation carriers (227).

Hence, before we can use miRNAs in the therapeutic setting, numerous obstacles remain to be overcome. The delivery method is the key impediment. We might be able to get over this obstacle with the use of chemical alterations, viral vectors, or nanoparticles. Despite these delivery issues, it is possible that miRNAs will play a significant role in cancer therapy, including BC, in the future. A novel approach to treating breast cancer that combines miRNA therapies with conventional chemotherapeutic techniques and drug targets is possible, but further study is needed before this promising paradigm can be implemented in the clinic. Thus, this review emphasizes how important it is to prioritize clinical trials and therapeutic interventions to advance the precision oncology movement’s goal of “curing” breast cancer.

## Author contributions

SS, RT conceived the study. SS drafted the manuscript. SS, HS, AS, SG, VH, RT revised the manuscript critically for important intellectual content. RT, HS provided important comments on the manuscript. All authors approved the final manuscript. All authors contributed to the article and approved the submitted version.

## Glossary

miRNAs: MicroRNAs
BC: breast cancer
LABC: Luminal A Breast Cancer
LBBC: Luminal B Breast Cancer
HER2+: Human Epidermal Growth Factor Receptor-2 Enriched Breast Cancer
TNBC: Triple-Negative Breast Cancer
MAPK: mitogen-activated protein kinase
PI3K: Phosphoinositide 3-Kinase
mTOR: Mammalian Target of Rapamycin
ER: Estrogen Receptor
Pol II: RNA polymerase II
pre-miRNAs: Precursor miRNAs
Exp5: Exportin-5
RISC: RNA-induced silencing complex
CMF: Cyclophosphamide
methotrexate: and 5-fluorouracil
NAC: Neoadjuvant chemotherapy
BCS: Breast Conservation Surgery
EBCTCG: The Early Breast Cancer Triallist’s Collaborative Group
LRR: Locoregional Recurrence
DFS: Disease-Free Survival
OS: Overall Survival
pCR: Pathological Complete Response
LN: Lymph node
ASCO: American Society of Clinical Oncology
EMT: Epithelial-Mesenchymal Transition
MiniPDXTM: Minimal Patient-Derived Xenograft
PTEN: Phosphatase and TENsin homolog
5-FU: 5-fluorouracil
MSCs-Exo: Mesenchymal Stem Cell-Derived Exosome
ID4: Inhibitor of Differentiation 4
CDA: Cytosine Deaminase
MCF-7/DTX: Docetaxel-Resistant MCF-7 BC cell line
DOX: Doxorubicin
MCF-7/EPB: Epirubicin-Resistant MCF-7 BC cell line
ADR: Adriamycin
EIF4E: Eukaryotic Translation Initiation Factor 4E
EFS: Event-Free Survival
BCL-2: B-cell Leukemia/Lymphoma 2 Protein
CCND1: Cyclin D1 protein
DCTD: dCMP Deaminase
HIF-1: Hypoxia-inducible factor-1
DTX: Docetaxel
EPB: Epirubicin
GCB: Gemcitabine
ct-miRNA: circulating miRNA
EMT: epithelial-mesenchymal transition
MDR1: Multidrug Resistance gene
P-gp: P-glyocoprotein
GST-π: glutathione S-transferase
MRP: multidrug resistance-associated protein
ABC: ATP-binding cassette transporters
BCRP: breast cancer resistance protein
EGFR: epidermal growth factor receptor
CSCs: Cancer stem cells
DDR: DNA damage repair
